# The Role of Complement Component C5a in the Pathogenesis of Diabetic Kidney Disease: A New Kid on the Block?

**DOI:** 10.7759/cureus.103067

**Published:** 2026-02-05

**Authors:** Virginia Geladari, Eleni Paschou, Achilleas Betsikos, Dimitrios Pallas, Nikolaos Sabanis

**Affiliations:** 1 Department of Internal Medicine, General Hospital of Trikala, Trikala, GRC; 2 Department of General and Family Medicine, Medical Unit of Giannouli, Larissa, GRC; 3 Department of Hepatology, Avicenne Hospital, Bobigny, FRA; 4 Department of Nephrology, General Hospital of Trikala, Trikala, GRC

**Keywords:** advanced glycation end-products, anaphylatoxin c5a, complement system, complement-targeting agents, diabetic kidney disease (dkd)

## Abstract

Diabetic kidney disease (DKD) is a serious complication of diabetes mellitus pandemic and raises awareness as it is considered a worldwide health burden with significant cardiovascular and all-cause morbidity and mortality leading to enormous social and economic consequences. The complex pathogenesis of DKD is multifactorial, involving hemodynamic, metabolic, inflammatory, and fibrotic pathways leading to progressive kidney damage. The significant overlap and dynamic nature of these interconnected pathways, alongside the activation of oxidative processes in different renal compartments, namely the glomeruli, vasculature, and tubulointerstitial space, have not yet been fully demystified; hence, DKD is a heterogeneous disease entity regarding its clinical manifestations, histopathology, and the rate of progression, making it difficult to develop effective therapies. However, recent research brings into focus the central role of complement activation in the development and progression of DKD.

Complement cascade activation leads to the formation of anaphylatoxin C5a, a potent inflammatory mediator, which serves as a powerful attractant for neutrophils, monocytes and macrophages, increases vascular permeability, promotes mast cell degranulation and tissue regeneration and modulates effectively innate and adaptive immune responses. Growing evidence supports the pivotal role of elevated C5a levels as well as their activated receptors in DKD progression through activation of inflammatory pathways, mitochondrial dysfunction and generation of harmful reactive oxygen species, as well as premature aging of renal tubular epithelial cells that exacerbates the vicious cycle of inflammation and fibrosis.

C5a/C5a receptors (C5aR) axis is assumed to be up-regulated early in the course of the disease and, interestingly, prior to overt clinical manifestations, contributing substantially to the progression of kidney inflammation, fibrosis and glomerulosclerosis, suggesting that it may be an early indicator of subclinical renal damage. Of note, tubular deposition of C5a has been correlated with the severity of renal damage and tubulointerstitial fibrosis in human biopsies, while elevated C5a levels in urine have been strongly associated with 10-year kidney failure risk. Also, increased C5a levels in urine have been identified as a valuable and accurate biomarker to stratify diabetic patients with DKD into rapid and slow progressors, implying that urine C5a levels are a reliable predictor of progression to end-stage kidney disease (ESKD).

In experimental studies, therapeutic inhibition of C5a-activated signaling pathways seems to mitigate the above detrimental processes. More specifically, genetic deletion or pharmacological inhibition of the C5a/C5aR axis ameliorates kidney injury, albuminuria, and fibrosis through restoration of mitochondrial function, decrease of reactive oxygen species and inflammatory pathways, and attenuation of tubular epithelial cells premature aging. Hence, C5a/C5aR axis inhibition has emerged as a promising therapeutic target given that current therapy cannot completely prevent DKD progression to ESKD in many patients. Therefore, in this narrative review, we aim to summarize the available data from clinical and preclinical studies that unravel the central role of anaphylatoxin C5a in DKD pathophysiology and turn the spotlight of drug discovery efforts on complement-targeted therapeutics.

## Introduction and background

Diabetic kidney disease (DKD) is a major microvascular complication noted in both diabetes mellitus type 1 (T1DM) and type 2 (T2DM) and raises concerns as it remains the leading cause of end-stage kidney disease (ESKD) worldwide [1-3]. In 2017, the prevalence of diabetes mellitus (DM) was nearly 425 million people worldwide and estimations report that this figure is expected to increase to 629 million by 2045, while 30-40% of patients with DM will develop DKD during their lifetime [4]. Thus, following decades of steadily increasing prevalence of DM, the absolute number of people with DM has grown dramatically, contributing substantially to a higher risk of cardiovascular events and premature death and causing enormous economic burden on the health care system [5]. Notably, the economic toll of diabetes is rising sharply during DKD progression to ESKD, and the average monthly expenditure for patients receiving long-term hemodialysis has been estimated at $12,299 in the United States [6,7].

Despite that DM and its complications are a global health threat requiring early detection and effective multimodal management, the current preventative approach for DKD remains a challenge mainly due to its complex pathogenesis. The pathophysiology of DKD is multifactorial involving various interrelated hemodynamic, metabolic, fibrotic and inflammatory pathways alongside the activation of oxidative stress that leads to progressive structural and functional kidney changes [8]. The dynamic nature of these interrelated pathways and the substantial overlapping of several activated pathomechanisms occur in almost all renal compartments, including glomeruli, vasculature, and tubulointerstitial space, leading to podocyte and endothelial injury, extracellular matrix accumulation, and fibrosis [8]. Decoding the underlying molecular mechanisms implicated in DKD pathogenesis has emerged as a challenging scientific field of persistent investigation. Recently, various research data have proven that immunometabolism and activation of the complement network play a fundamental role in DKD pathophysiology [1,3,9].

The complement pathway is one of the most crucial pillars of the innate immune system, and complement-mediated kidney injury has been proven to be involved in the pathogenesis of several kidney diseases beyond atypical hemolytic uremic syndrome and C3 glomerulopathy, such as IgA nephropathy, membranous nephropathy, and DKD [3,10,11]. The metabolic and hormonal disorders of diabetes mellitus, mainly hyperglycemia, dyslipidemia and insulin resistance affect immune cells metabolism triggering the diversion of anti-inflammatory to pro-inflammatory intracellular processes, which in turn contribute to the development of diabetes complications and DKD [9].

More specifically, hyperglycemia leads to increased intraglomerular pressure and hyperfiltration through tubuloglomerular feedback dysregulation caused by decreased delivery of sodium to the macula densa in response to increased reabsorption of glucose and sodium in the proximal tubule. Hyperfiltration of proteins to the tubular lumen leads to increased tubular reabsorption of them, which eventually promotes the release of pro-inflammatory and pro-fibrotic molecules predisposing to irreversible nephron damage. This pathway is further exacerbated due to imbalance of vasoactive molecules, mainly angiotensin II and endothelin-1 [12]. Simultaneously, under hyperglycemic conditions, several metabolic pathways are activated, including hexosamine pathway, polyol pathway, and protein kinase C (PKC) pathway, orchestrating the production and accumulation of reactive oxygen species (ROS) that oxidize several important macromolecules. Advanced glycation end products (AGEs) pathway is also activated in a hyperglycemic microenvironment, contributing substantially to ROS accumulation alongside its central role in complement activation, which results in significant tissue damage through inflammatory and fibrotic signaling pathways [13].

Proteomic analysis in diabetic kidney biopsy samples has confirmed the contribution of complement system activation in DKD through the accumulation of complement components in affected kidneys [14]. More than that, urinary complement proteome analysis has been recently linked to DKD progression, and C5a has been identified as a strong predictor of kidney failure within ten years of follow-up [15]. In the same study, the increased expression of related genes in kidney tissue was indicative of local complement cascade activation rather than systemic complement activation, which could simply result in complement protein leakage into the urine [15]. High urine complement protein levels, including C5a, have been also emerged as powerful and independent biomarkers to identify rapidly progressive DKD and stratify DM patients with DKD into rapid and slow progressors [16].

The three complement pathway systems are activated in DKD, ultimately converging to anaphylatoxin C5a formation [2,3,17,18]. Of note, elevated levels of C5a in patients with diabetes and in experimental models precede the appearance of DM-associated clinical manifestations [17]. The anaphylatoxin C5a is a potent inflammatory mediator that serves as a powerful chemoattractant recruiting neutrophils, monocytes, macrophages, and T-lymphocytes, and activating them to release granular enzymes and to produce various other inflammatory mediators such as cytokines and chemokines. Moreover, anaphylatoxin C5a affects vascular permeability facilitating leucocyte infiltration and promotes mast cell degranulation and tissue regeneration and modulates innate and adaptive immune responses through actions on T-cell differentiation and Fc receptor expression on leukocytes [19].

Thus, C5a-dependent recruitment of inflammatory cells promotes the infiltration of kidney tissues by monocytes and macrophages, which are stimulated to release pro-inflammatory cytokines and produce high levels of transforming growth factor-beta (TGF-β), leading to glomerular damage and tubulointerstitial fibrosis, respectively [20]. More specifically, complement activation and macrophage phenotype have been closely linked leading to a cycle of chronic inflammation and renal fibrosis. In early stages of DKD, a pro-inflammatory phenotype of macrophages is the predominant cell type driving inflammatory processes, while in the advanced stage of the disease, an anti-inflammatory but pro-fibrotic macrophage phenotype orchestrates kidney fibrosis through transformation into myofibroblasts that release massive amounts of TGF-β [21,22].

The powerful biologic activities of anaphylatoxin C5a and inflammatory sequelae occur through the C5a/C5aR axis activation that initiates G-protein coupled signaling pathways and has also been incriminated for disrupting mitochondrial metabolism and exacerbating oxidative stress resulting in injurious kidney effects [17]. Apart from mitochondrial damage and generation of harmful reactive oxygen species (ROS), C5a/C5aR axis upregulation has been correlated with premature aging of renal tubular epithelial cells through activation of p53/p21 and NF-κB pathways, which are involved in cell cycle arrest and a state of chronic inflammation, respectively [23].

Hence, under hyperglycemic conditions, complement activation induces tubular cell senescence through upregulation of C5a/C5aR axis. Ultimately, senescent epithelial cells express a senescence-associated secretory phenotype and release pro-inflammatory cytokines that exacerbate the vicious cycle of inflammation and fibrosis, while concomitantly, the expression of anti-aging protein Klotho is down-regulated [24]. Noteworthy, the hyperactivation of complement component C5a in DKD diabetic mice incites the above inflammatory cycle through disruption of the gut-kidney axis that eventually accelerates kidney damage. This interaction has been correlated with the adverse effects of C5a hyperactivation on the permeability of monolayers of intestinal epithelial cells through activation of the ERK pathway and production of several pro-inflammatory cytokines, as well as reduced expression of claudin-1, an essential protein that maintains gut permeability. Concomitantly, C5a hyperactivation induces gut dysbiosis and reduces short-chain fatty acid (SCFA) production that in turn plays a fundamental role in regulating the immune response and inflammatory cascade in the host. In contrast, C5 blockade seems to attenuate renal inflammation and partly restore the disrupted gut-kidney axis suggesting that complement component C5 is a potential therapeutic target for the treatment of DKD [25]. 

Herein, in this narrative review, we collected published literature related to the activation of complement system in the pathogenesis of DKD up to October 2025, focusing on the role of complement component C5a and C5a/C5aR axis activation in DKD pathogenesis as well as its potential significance as an early DKD biomarker and a therapeutic target. For the purposes of the review, a thorough search of the current literature was conducted in PubMed using the following key words: “complement system activation” OR “complement cascade activation” OR “complement component C5a” OR “complement C5a/C5aR axis activation” AND “diabetic kidney disease” OR “diabetic nephropathy”. Literature that was consistent with the topic of interest was used as a reference. Article types published in English included original manuscripts and reviews, and in the selection process, two independent authors were involved who performed the search and merge separately.

Complement system activation and complement cascade inhibitors

The complement system is a family of approximately 50 soluble interacting and interactivating proteins including their receptors and it is considered one of the most potent and well-preserved innate mediators of immune system [1-3,18]. Traditionally, the complement cascade activation occurs through three major pathways: the classical, the mannose-binding lectin (MBL), and the alternative pathway with distinct instigators and inhibitors [1,3,18].

**Figure 1:**
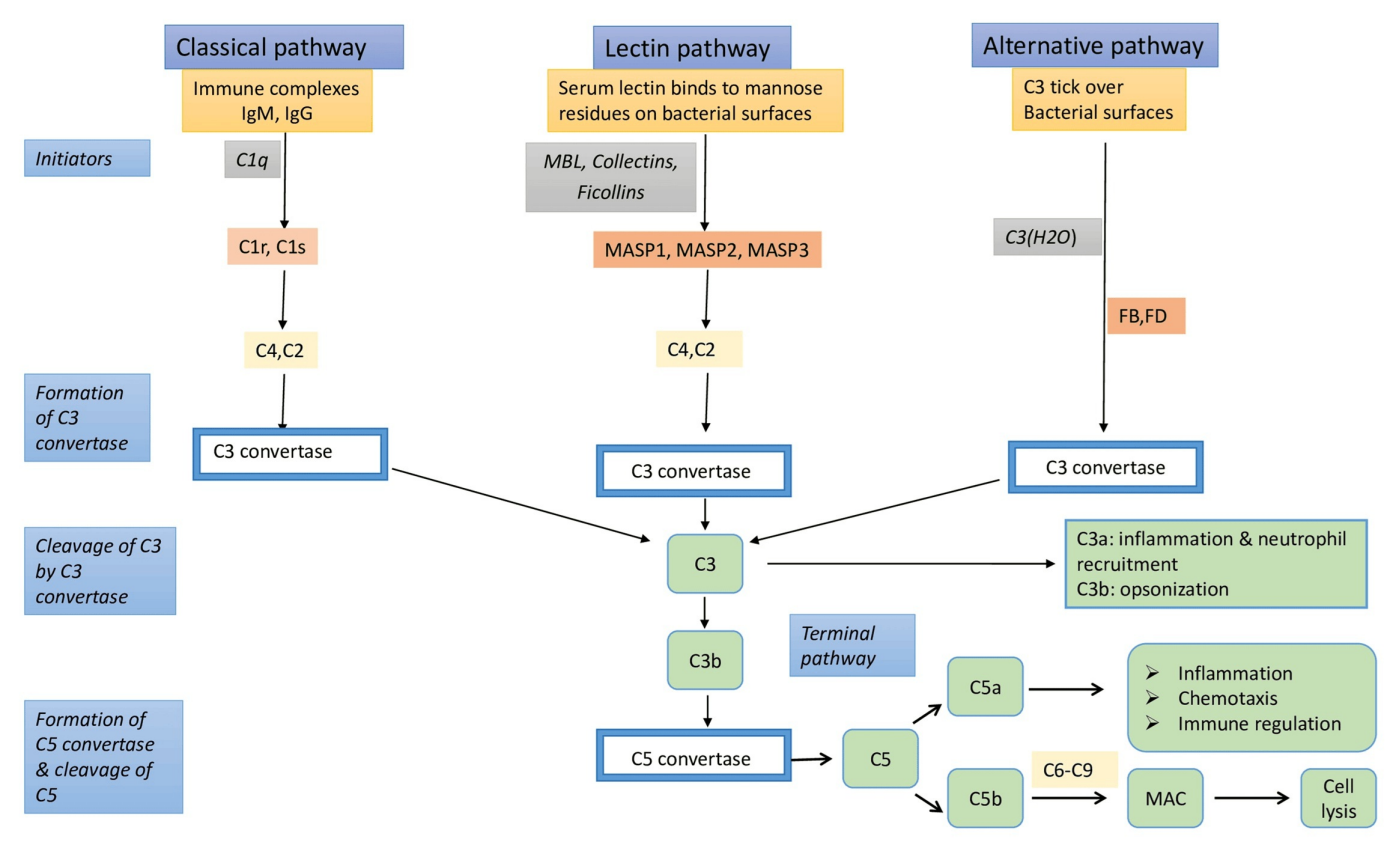
Complement cascade activation occurs through three major pathways: the classical, the mannose-binding lectin (MBL), and the alternative pathway. The complement system is a family of approximately 50 soluble interacting and interactivating proteins. Traditionally, the complement cascade activation occurs through three major pathways: the classical, the mannose-binding lectin (MBL), and the alternative pathway with distinct initiators. Classical pathway stimulation is typically mediated via immune complexes, alternative pathway activation is mainly characterized by spontaneous C3 hydrolysis at a tick-over manner that is further triggered by multiple carbohydrate molecules, and lectin pathway activation is triggered through recognition of glycated proteins or pattern recognition molecules (PRMs), such as ficolins, leading to activation of mannose-associated serine proteases. Regardless of the primary stimulus, all complement pathways share a common endpoint: cleavage of components C3 and C5, causing inflammation through production of anaphylatoxins C3a and C5a, opsonization via C3b component and cell damage and lysis through the formation of the membrane attack complex (MAC) assembled by the polymerization of the terminal complement components (C5b and C6-C9). The above schematic representation was designed by Nikolaos Sabanis and Virginia Geladari with Microsoft PowerPoint 2021 Windows. DAF: decay-accelerating factor; MCP: membrane cofactor protein, MBL: mannose-binding lectin, MASP: mannose-associated serine proteases.

Classical pathway stimulation is typically mediated via immune-complexes associated activation of C1 compound, while alternative pathway activation is mainly characterized by spontaneous C3 hydrolysis at a tick-over manner that is further triggered by multiple carbohydrate molecules [1,3,18]. On the other hand, the leading force of lectin pathway activation is considered to be the recognition of glycated proteins or pattern recognition molecules (PRMs), such as ficolins, by MBL leading to activation of mannose-associated serine proteases (MASPs) [1,3]. Regardless the primary stimulus, all complement pathways share a common endpoint: cleavage of components C3 and C5, causing inflammation through production of anaphylatoxins C3a and C5a, opsonization via C3b component and cell damage and lysis through the formation of membrane attack complex (MAC) assembled by the polymerization of the terminal complement components (C5b and C6-C9) [1,3,18] (Figure 1).

**Figure 2:**
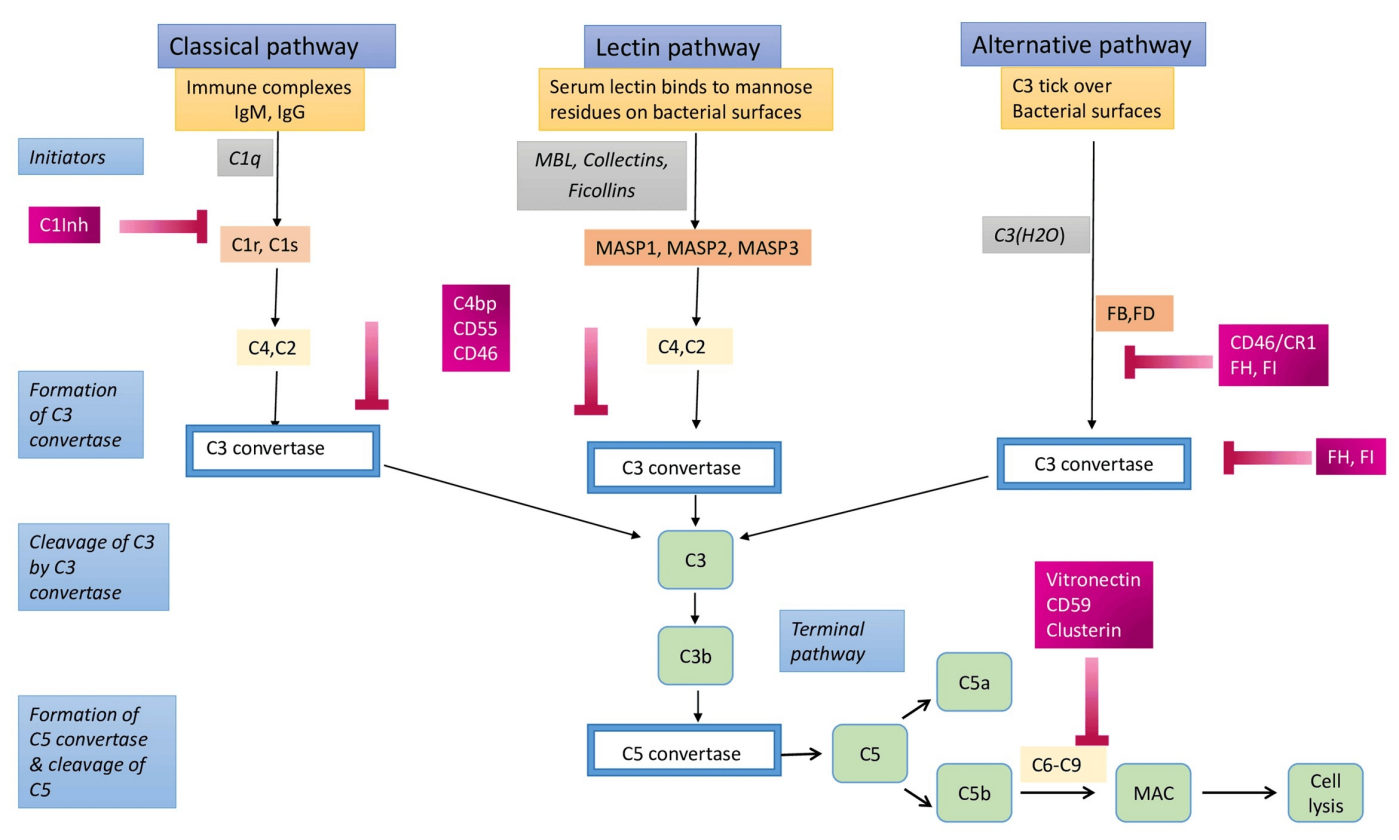
The complement cascade inhibitors. Permanent downstream stimulation of the complement system is further regulated by several inhibitors, including C1INH, MCP, CD59, DAF, factors H and I, acting in critical checkpoints to avoid uncontrolled activation. The schematic representation of complement cascade initiators and inhibitors was created by Nikolaos Sabanis and Virginia Geladari with Microsoft PowerPoint 2021 Windows. DAF: decay-accelerating factor; MCP: membrane cofactor protein, MBL: mannose-binding lectin, MASP: mannose-associated serine proteases.

Permanent downstream stimulation of the complement system is further regulated by several inhibitors such as C1INH, membrane cofactor protein (MCP), CD59, decay-accelerating factor (DAF), factors H and I, acting in critical checkpoints to avoid uncontrolled activation [1,3,11] (Figure 2).

Nowadays, growing evidence suggests a remarkable involvement of the complement system in the pathogenesis of acute and chronic kidney diseases through clinical and experimental data [11,18]. The recognition of complement components in kidney biopsy samples in several kidney diseases, including DKD, the correlation between mutations in complement proteins and kidney diseases through genetic analysis, evidence of local expression of complement components in tubular, glomerular and mesangial cells and urine isolation of complement fragments advocates for increased susceptibility of kidneys to the complement-mediated injury [11,18].

Molecular origin and structure of C5a

The gene of complement compound C5 is located in the human chromosome 9, in position 9q33.2, and its size measures approximately 79kb [26]. Its expression produces protein C5, which is primarily synthesized as a pre-protein (pro-C5) consisting of an α-chain and a β-chain linked together by a disulfide bond and containing 1676 amino acid residues [27,28]. This precursor protein undergoes several modifications in order to obtain a mature form [27, 28]. The regions that structure the molecule of C5 include 8 alpha2-macroglobulin (MG1-8) like domains, a C5d domain (thioester containing domain), a C5a or anaphylatoxin domain, a complement C1r/C1s, Uegf, Bmp1 (CUB) domain, and a C345C domain in the C-terminal arm of a-chain [27,28].

The proteolytic cleavage of the component C5 by C5 convertases occurs in a specific site in the α-chain of the molecule (between R751 and L752) and consists a key point in the activation of the terminal complement cascade [27,28]. This splitting leads to the production of two significant terminal fragments, a smaller C5a and a larger C5b [27]. After cleavage, the C5b fragment undergoes further structural alterations in order to increase affinity with component C6 and finally interacts and polymerizes with C6, C7, C8, and multiple C9 complement proteins, generating the membrane attack complex (MAC or C5b-9 complex), which contributes to the formation of lytic pores and destruction of pathogen cells [27,28].

The 74 amino acid-containing C5a fragment is demonstrated as a four helical formation, with a bundled core structure in a drumstick shape, held by three disulfide bonds [26,28]. C5a is considered to act as the most efficacious complement anaphylatoxin [26-28].

More specifically, it is supposed to manifest both spasmogenic and chemotactic functions resulting in systole of smooth muscles, histamine release from mast degranulation, production of ROS, vasodilation, increased vascular leakage, chemoattraction of innate and adaptive immunity cells and cytokine and interleukin storm stimulation [26-28]. It is also recognized from almost all myeloid cells; however, its physiological effect has been expanded in numerous other cells, such as kidney tubular, endothelial, microglia, nerve, hepatic and vascular cells [26,28]. New evidence reinforces the role of C5a as a strong inflammatory mediator in the pathogenesis of multiple immune- and non-immune-mediated diseases characterized by complement activation [26,27].

C5a regulation and activation

As every potent molecule, C5a remains in systemic circulation for approximately three to five minutes and its function is strictly controlled by several regulatory proteins [26]. One of the most well-known regulatory enzymes of C5a is carboxypeptidase N (CPN), which in high concentrations quickly converts C5a to C5adesArg74 by removing the C-terminal arginine residue [26,27]. C5adesArg74 is a less efficient mediator than C5a with inferior anaphylactic and chemotactic activity, and it is supposed that this derivative binds C5a receptors with slightly altered affinity than C5a [26,27].

Anaphylatoxin C5a performs its functions through linkage with two different types of receptors, C5aR1 and C5aR2 (or C5L2), which are located and expressed in several immune and non-immune cell populations [18,26,27]. C5aR1 and C5aR2 are homologous in approximately two-thirds of their sequences, and both consist of seven-transmembrane protein receptors belonging in class A rhodopsin-like receptor subfamily [18,26,27]. However, they differentiate in cell localization and signal transmission, as C5aR1 is mainly an extracellular G-protein coupled receptor (GPCR), whereas C5aR2 is an intracellular receptor, which is generally not interacting with G-proteins and typically couples β-arrestins [18,26,27]. Receptors are being activated from both C5a and C5adesArg74 [26]. Affinity for C5a is considered to be similar amongst the two receptors; however, affinity of C5a desArg74 ranges and seems to be diminished towards C5aR1 when compared with C5a, and as for C5aR2, data support similar compatibility [26,27]. High C5a concentration also provokes the formation and activation of C5aR1/C5aR2 heterodimers leading to stimulation of ERK 1/2 signaling pathway and C5aR1 internalization [26,27].

More specifically, complement component C5a primarily induces its anaphylactic and chemotactic functions throughout binding to C5aR1 [26,27,29]. The binding of C5a with this GPCR causes the linkage of the Gα subunit, followed by the conversion of guanosine diphosphate (GDP) to guanosine triphosphate (GTP) in this domain [26,27,29]. Further release of the dimer Gβγ and the Gα-GTP complex stimulates several signaling pathways, such as PI3 kinases, protein kinase C, phospholipase D, and also mediates intracellular elevation of calcium levels, causing activation of mitogen-activated protein kinase (MAPK)/extracellular signal-related kinase (ERK), which ultimately results in progression of pro-inflammatory reactions [26,27,29]. Simultaneously, activation of C5a/C5aR1 axis is associated with the expression of transcription factors, nuclear factor-κβ (NF-κβ), mitochondrial dysregulation and production of ROS, release of pro-inflammatory cytokines and stimulates the activation of nucleotide-binding oligomerization domain (NOD)-leucine-rich pyrin-domain containing 3 (NLRP3) inflammasomes [26,27,29].

In greater detail, it has been shown that C5a/C5aR1 axis activation interferes with mitochondrial homeostasis of tubular epithelial cells through alterations in mitochondrial architecture, bioenergetics and fatty acid profile, like cardiolipin remodeling. Thus, mitochondrial dysfunction and disrupted mitochondrial metabolic agility lead to increased ROS production, altered mitochondrial respiration, fibrosis and cellular damage [17]. Moreover, C5a/C5aR1 axis activation has been implicated in diabetes-induced cell cycle arrest and tubular epithelial cell senescence. Coughlan et al. discovered that administration of valproic acid in streptozotocin-induced diabetic mice led to a significant reduction in albuminuria and glomerulosclerosis through C5a receptors downregulation concomitantly with a decrease of cellular senescence markers. In the same study, similar results were observed in diabetic mice after administration of the C5aR1 inhibitor, PMX53, concerning the genes associated with cell cycle pathways leading to cellular senescence [30].

Immunohistochemistry and ligand-binding studies demonstrated that C5a receptors are expressed in a variety of not only myeloid cells like macrophages, monocytes, neutrophils, dendritic cells, but also in non-immune cells, such as vascular, lung, hepatic and neuron cells [29]. Interestingly, growing data suggest advanced expression of anaphylatoxin C5a, C5aR, and their mRNAs in kidneys, especially in mesangial, proximal tubular cells, podocytes, fibroblasts, vascular endothelial, and smooth muscle cells [1,26,31,32]. Upregulation of these receptors in kidney cells explains the C5a-mediated renal injury, which is observed in several diseases, such as DKD, and it is going to be analyzed below [1,32].

On the contrary, the role of C5aR2 has been strongly disputed, with evidence supporting both anti-inflammatory and pro-inflammatory contributions [33]. The expression of these questionable receptors takes place in a wide variety of myeloid and non-myeloid cell populations, including kidney cells, similarly to C5aR1 [33]. C5a/C5aR2 axis activation is known that inhibits ERK pathway and therefore inducing anti-inflammatory responses by acting as a decoy receptor [26,33]. In support of the above, Zhao et al. found that genetic deletion of C5aR2 in diabetic mouse models markedly aggravated the DKD phenotype. In the same study, combining lipidomic and transcriptomic analyses revealed that C5aR2 deletion induced mitochondrial dysfunction and endoplasmic reticulum (ER) stress, whereas treatment with the C5aR2-specific agonist P59 improved mitochondrial and ER function through upregulation of phosphatidylserine (PS) synthases [34]. Similarly, Trambas et al. showed that deletion of C5aR2 in diabetic mice did not have a significant effect on inflammatory genes, such as MCP-1 and tumor necrosis factor-alpha (TNF-α), albuminuria, and renal fibrosis [35].

**Figure 3:**
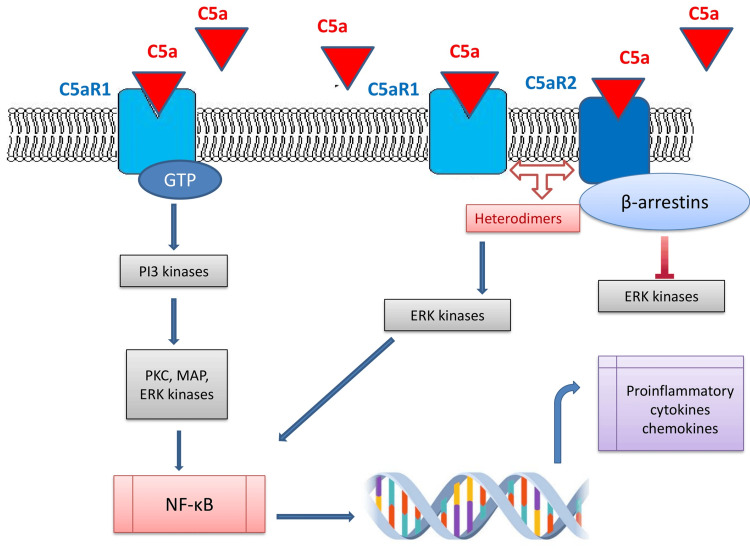
C5a anaphylatoxin biological actions through binding with two different types of receptors, C5aR1 and C5aR2. C5a performs its actions through linkage with two different types of receptors, C5aR1 and C5aR2. C5aR1 and C5aR2 are seven-transmembrane protein receptors belonging in class A rhodopsin-like receptor subfamily. C5aR1 is an extracellular G-protein-coupled receptor, whereas C5aR2 is an intracellular receptor not interacting with G-proteins, which typically couple with β-arrestins. C5a primarily induces its anaphylactic and chemotactic functions throughout binding to C5aR1. Specifically, C5a/C5aR1 activation stimulates several signaling pathways, such as PI3 kinases, protein kinase C, phospholipase D, and also intracellular elevation of calcium levels, causing activation of mitogen-activated protein kinase (MAPK)/extracellular signal-related kinase (ERK), which ultimately results in progression of pro-inflammatory reactions. Moreover, activation of the C5a-C5aR1 axis promotes the expression of transcription factors, nuclear factor-κβ (NF-κβ), mitochondrial dysregulation and production of ROS, release of pro-inflammatory cytokines, and stimulates the activation of NOD-leucine-rich pyrin-domain containing 3 (NLRP3) inflammasomes. On the contrary, C5aR2 activation is known that inhibits ERK pathway and therefore inducing anti-inflammatory responses. However, high C5a levels provoke the formation and activation of C5aR1/C5aR2 heterodimers leading to stimulation of the ERK 1/2 signaling pathway resulting in the production of pro-inflammatory mediators, such as cytokines and interleukins. The above schematic representation was designed by Nikolaos Sabanis and Eleni Paschou with Microsoft PowerPoint 2021 Windows. PKC: protein kinase C, GTP: guanosine triphosphate.

However, other studies have revealed its central role in inflammatory processes through the formation of heteromers with C5aR1. As a result, ERK and microtubule affinity-regulating kinases (MARK) signaling pathways are activated leading to the production of pro-inflammatory mediators such as cytokines and interleukins [26,33]. Also, in experimental models, C5a/C5aR2 interaction seems to contribute to NLRP3 inflammasome activation, protein kinase R expression, and pro-inflammatory protein high-mobility group box 1 (HMGB1) release from macrophages. On the contrary, in C5aR2-deficient models, NLRP3 activation and HMGB1 release are restricted due to inhibition of cytokine maturation and inflammatory responses [36] (Figure 3).

The role of immunometabolism in diabetic kidney disease

Theoretically, DKD is mainly a metabolic and vascular disorder originated from interdependent alterations caused by diabetes-affecting molecules, namely DNA, proteins, and lipids [10,37]. Although DKD is not yet characterized as an immune-mediated disorder, certain intracellular and extracellular metabolic modifications stimulate the activation of immune responses orchestrating the progression of DKD to advanced stages [9,37-39]. Under this spectrum, immunometabolism is strongly correlated with DKD through metabolic reprogramming in both innate and adaptive immunity [9].

More specifically, the high immune activity in DKD implicates several interrelated mechanisms, mainly the altered metabolic pathways and hemodynamic changes. The altered metabolic milieu, including hyperglycemia, dyslipidemia, insulin resistance, pancreatic β-cell depletion, and mitochondrial dysfunction, results in release of reactive oxygen species (ROS) and creation of advanced glycated end products (AGEs) alongside hemodynamic changes due to the overactivation of renin-angiotensin-aldosterone system (RAAS). The above mechanisms maintain and inflame a vicious cycle of activation of immune responses through damage of kidney cells and release of damage-associated molecular patterns (DAMPs), pro-inflammatory cytokines, and chemokines [38].

In particular, RAAS activation is a central player of DKD progression leading to increased intraglomerular pressure, renal inflammation, generation of ROS, and kidney fibrosis through increased local intrarenal activity of angiotensin II rather than systemic RAAS activation. Apart from glomerular capillary hypertension that results in damage to podocytes, endothelial and mesangial cells, angiotensin II and aldosterone exert important pleiotropic effects, including extracellular matrix accumulation, endothelial dysfunction, adhesion molecules expression, and up-regulation of NF-κΒ and TGF-β pathways [40].

The noxious effects of these alterations on endothelium, podocytes, mesangial and immune cells orchestrate the accumulation and infiltration of macrophages, monocytes, and T-cells, the continuous expression of cytokines and adhering molecules, as well as the activation of the complement system [10,38]. Thus, further progression of DKD occurs leading to tubulointerstitial fibrosis, glomerulosclerosis, and modifications in kidney architecture [10,38,39].

Mechanisms of complement activation in diabetic kidney disease

Chronic hyperglycemia plays a central role in complement-mediated kidney injury in DKD. Under hyperglycemic conditions, significant chemical and structural alterations occur through glycation in several molecules, such as proteins, lipids, and nucleic acids, leading to the formation of advanced glycation end products (AGEs) [1,3,18].

In this setting, complement activation is mainly attributed to the following mechanisms [1,3,18]. The first mechanism is considered to be the activation of the lectin pathway through distinguishing new epitopes by MBL as a result of the advanced protein glycation [3,41]. Moreover, Fortpied et al. revealed a strong pathophysiological link between the fructosamine pathway and lectin pathway activation in diabetes through MBL binding to fructoselysine, accompanied with complement activation [42]. The second implicated mechanism is associated with the chronic hyperglycemia-induced dysregulation of the inhibitory complement machinery leading to weak control of the complement cascade overactivation. In particular, CD59 glycoprotein, a potent inhibitor of the complement membrane attack complex (MAC), undergoes non-enzymatic glycation to a structurally modified and functionally inactive form. CD59 inactivation leads to MAC accumulation, inducing cytotoxicity, inflammation, and thrombosis [43]. In the same context, glycation-induced impairment of complement alternative pathway (AP) regulators, including complement factor B (CFB), complement factor H (CFH), CFHR1, CFHR3, and CFHR5, has been correlated with the dysfunction of their regulatory activity, leading to AP overactivation [44]. The third mechanism refers to the activation of the classical pathway through the binding of the C1q complement component by the superabundance of AGEs [1,3,18,45].

**Figure 4:**
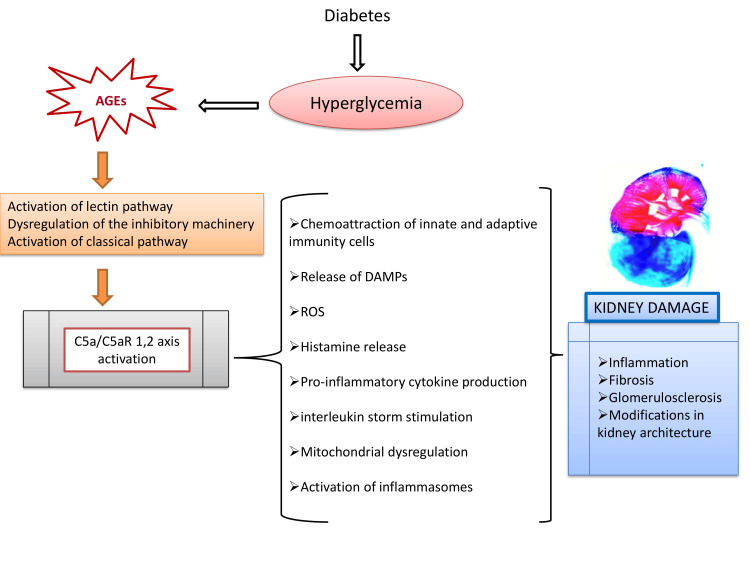
Complement cascade activation orchestrates DKD progression through multiple interconnected pathways. Chronic hyperglycemia plays a central role in complement-mediated kidney injury in DKD. Under hyperglycemic conditions, significant chemical and structural alterations occur through glycation leading to the formation of advanced glycation end products (AGEs). In this setting, complement activation is mainly attributed to the following mechanisms: 1. Αctivation of the lectin pathway through distinguishing new epitopes by MBL as a result of the advanced protein glycation; 2. High glucose-induced dysregulation of the inhibitory complement machinery leads to weak control of the complement cascade overactivation; 3. Activation of the classical pathway through the binding of the C1q complement component by the superabundance of AGEs. Hence, once the complement cascade pathway is activated, formation of anaphylatoxins (C3a and C5a), MAC, and release of pro-inflammatory mediators intensify the DKD progression through inflammatory and fibrotic signaling pathways. The above graphical representation was created by Nikolaos Sabanis with Microsoft PowerPoint 2021 Windows. DKD: diabetic kidney disease; DAMP: damage-associated molecular patterns; ROS: reactive oxygen species; MBL: mannose-binding lectin.

Hence, once the complement cascade pathway is activated, formation of anaphylatoxins (C3a and C5a), MAC, and release of pro-inflammatory mediators orchestrate DKD progression through multiple interconnected pathways [1,3,11] (Figure 4).

## Review

Evidence of complement involvement in diabetic kidney disease

Till now, several clinical studies have described etiological correlations between complement protein detection in human samples and progression of DKD by using minimally invasive or invasive methods. For example, high serum concentrations of MBL and H-ficolin have been correlated with the development and severity of albuminuria and may predict the long-term progression of DKD [1,46,47]. In support of the above, renal local MBL pathway activation has been proposed to contribute to renal interstitial damage in type 2 diabetic nephropathy patients [48]. Furthermore, C3c deposition, a downstream product of MBL as well as an indicator of alternative pathway overactivation, has been emerged as an independent predictor for end-stage renal disease (ESRD) in DKD patients rather than C1q deposition, indicative of classical pathway activation [49].

The role of C3, as a disease predictor, remains controversial as there is evidence for both elevated and decreased serum values. More specifically, elevated plasma C3 levels were associated with a high risk of diabetic complications and reported as robust predictors of non-diabetic kidney diseases, while decreased levels were connected with aggregated histological kidney damage and renal impairment in patients with T2DM [1]. Several complement proteins and activation products, including C1q, C4d, C5, C5a, C3a, MBL, Bb, and MAC, were elevated in serum and urine samples of DKD patients, indicative of an activated complement network in DKD [1].

In detail, urinary abundance of complement proteins C3, C5, CD59, C9, and complement factor H was significantly correlated with kidney failure in patients with T2DM and biopsy-proven DKD [50]. Similarly, several peptides derived from the complement proteins C3, C4, and complement factor B in urine were found to be significantly associated with specific kidney disease etiologies, including DKD [51], while high MBL concentrations in urine were associated with high mortality rates in T1DM compared with low MBL concentrations in a 12-year follow-up study [52]. Moreover, urine proteomic analysis in both T1DM and T2DM patients pointed out a positive correlation between complement factor C5 and its fractions, C5a and C5b, and the presence of albuminuria [1]. Recently, urinary proteome analysis in two prospective cohorts with type 1 and 2 diabetes and advanced DKD was strongly linked to DKD progression. Among others, urinary proteins of the complement system, C5a, C2, C7, and CFH were robustly associated with 10-year kidney failure risk independently of clinical covariates. Notwithstanding, the above associations were especially pronounced in advanced kidney stages and far stronger for urinary than circulating proteins, indicative of local activation of the complement network [15].

In terms of plasma levels of complement proteins, patients with DKD had significantly higher plasma levels of C1q, MBL, C3a, C5a, C4d, and sC5b-9 complement-derived proteins than patients with diabetes only [53]. Similarly, the levels of C7 in serum were increased in early diabetic kidney disease patients compared to healthy controls, suggesting that C7 can be used as a molecular target for the detection of early DKD [54].

Genes associated with activation of complement pathways revealed being up-regulated, while the inhibitory ones were downregulated in glomerular and tubular cells throughout gene-wide transcriptome analysis in diabetic biopsy specimens [1,55]. Woroniecka et al. identified in human diabetic kidney biopsy samples a six-fold increase in gene expression of C3 in glomeruli tissue compared with healthy individuals [50]. In the same context, Yang et al. analyzed the glomerular proteome of complement proteins in kidney biopsies from 40 DKD patients and detected a critical role of classical and alternative pathways of complement rather than the lectin pathway in disease progression [44]. More specifically, glomerular complement proteins of classical and alternative pathways were positively associated with glomerular pathological lesions and proteinuria, while negatively associated with eGFR [44].

Sun et al. investigated renal biopsy specimens from 161 DN patients using direct immunofluorescence, light and electron microscopy, and revealed a clear association between complement deposition of C1q and C3c with more severe kidney damage, including higher scores of interstitial fibrosis and inflammation, tubular atrophy, and vascular lesions, as well as significantly higher levels of urinary protein [56]. Additionally, immunohistochemistry in DKD samples revealed positive staining for C5a in tubular cells, indicative of complement cascade activation during disease progression [1]. Of note, complement C5a and C5aR were coexpressed in glomerular endothelial cells of kidney biopsy samples, indicative of a complex interplay among complement activation, endothelial dysfunction, and progression of diabetic glomerulosclerosis [25].

The above findings consist of strong evidence that complement activation not only plays a significant role in DKD pathogenesis but also its protein compounds, such as C5a, may be valuable disease biomarkers, predictors, and potential therapeutic targets.

C5a and kidney injury in diabetic kidney disease

As mentioned above, complement activation gains ground in the pathogenesis of diabetic nephropathy mainly through the uncontrolled production of the potent anaphylatoxin C5a. The overactivation of the C5a/C5aR axis is considered to be a central position player concerning initiation and progression of kidney injury, inflammation, and fibrosis [1,26].

Several studies examined the C5a plasma levels in both T1DM and T2DM patients and animal models. Results pointed out a significant increase in C5a plasma levels in normoalbuminuric experimental diabetic models, suggesting that activation of the C5a/C5aR1 axis might be a harbinger of diabetes complications and DKD [17,18]. Renal C5a/C5aR axis activation is assumed to induce inflammation, kidney damage, and fibrosis through chemoattraction and infiltration by macrophages, neutrophils, and adaptive immunity cells, production of ROS, pro-inflammatory and pro-fibrotic cytokines, and renal proliferation of fibroblasts [17,18,57]. Therefore, the activated C5a/C5aR1 axis substantially contributes to the initiation and acceleration of kidney damage and tubulointerstitial fibrosis [18,57]. In support of the above, preclinical studies in mice revealed that targeting and blocking the C5a/C5aR1 signaling pathway resulted in reversal of inflammatory processes, fibrosis, and glomerulosclerosis [17,18].

Moreover, studies in patients with diabetes mellitus revealed a positive association between plasma levels of C5a and urine albumin excretion and also a reverse correlation with the estimated glomerular filtration rate (eGFR). However, these findings did not prove to have statistical significance [17]. Certainly, in other studies, researchers observed that during the progression of diabetic nephropathy, the altered urine complement concentrations and immunohistochemical depositions were positively correlated with renal tubular injury and ESKD [50,58].

Specifically, elevation in C5a urine expression and staining for C5aR1 proved to be positively associated with advanced diabetic nephropathy class and progression of tubular fibrosis and damage, while a similar statistical correlation was observed with proteinuria, serum creatinine levels, and eGFR decline [58].

In experimental diabetic mice and rat models, transcriptome analysis has also proven C5a and C5aR1 overexpression in the renal cortex. These results may reflect not only systemic C5a/C5aR1 axis upregulation but also significant local kidney C5a overproduction [17]. Indeed, the cortical overactivity of C5a and its receptors was observed early in the onset of diabetes and, interestingly, prior to the occurrence of renal complications and proteinuria [17]. Urine examination and proteomic analysis confirmed that elevated C5a levels were an independent risk factor for the development of diabetes-associated injury [1,17]. Furthermore, increased urine C5a levels were mainly noticed in advanced diabetes and remained high during the progression of the disease [17]. Additionally, urinary excretion of C5a was proven to be an excellent biomarker of histological progression of DKD in humans, with almost 80% sensitivity and approximately 70% specificity to predict DN in advanced stages (III and IV) [58].

Interestingly, important conclusions were drawn from genetic and pharmacological C5a/C5aR1 silencing interventions. Particularly, in genetically modified experimental diabetic models, the deletion of C5aR1 resulting in a significant decline in albuminuria (>75%) [17]. Oxidative stress and inflammatory biomarkers, as well as immune cell infiltrations, were also significantly reduced, demonstrating a clear causal relationship between C5a/C5aR1 pathway activation and evolution of diabetic nephropathy, while targeting this axis led to substantial nephroprotection [17,18].

Similarly, administration of C5a inhibitors or C5a receptor antagonists (C5aRAs) showed appreciable reduction in albuminuria and important decline in inflammatory chemokines production [17]. Furthermore, in experimental models after treatment with C5aRAs, the histological findings revealed improvement in glomerular hypertrophy and considerable decrease in mesangial proliferation, kidney infiltration by neutrophils, and basement membrane thickening [25,32]. In the same context, transcriptomic analyses and metabolomics on renal samples showed rehabilitation in expression of genes that participate in mitochondrial dysfunction and metabolic dysregulation both in diabetes mellitus and in diabetic kidney disease [17].

New therapeutic approaches

Understanding the causal relationship between DKD initiation and progression and complement overactivation has led to continuous investigation in the field of complement blockade. Taking into account the kidney vulnerability to C5a deleterious effects, it seems insightful that complement-targeted therapeutics could offer significant benefit.

Eculizumab, a humanized monoclonal antibody against C5, which prevents its conversion to C5a and C5b fragments, was the first drug targeting the complement cascade authorized for clinical use in several diseases, including complement-mediated kidney diseases [18]. Following that, various similar anti-C5 drugs were released, such as ravulizumab and crovalimab. Although its revolutionary role in targeting complement, eculizumab and other anti-C5 inhibitors share the same difficulty in everyday clinical practice as they are listed among the more expensive and inaccessible therapies, especially in resource-limited settings [1,18]. Furthermore, patients treated with these drugs are susceptible to several infections due to inhibition of MAC formation [18]. In particular, C5 inhibitor therapy substantially increases susceptibility to invasive meningococcal disease, as well as other pathogens, such as *Streptococcus pneumonia* and *Haemophilus influenzae*, given that MAC formation is necessary to kill encapsulated bacteria. Thus, mandatory vaccinations are required or combined protection with vaccinations and continuous antibiotic prophylaxis [59].

For that reason, the discovery of selective C5a axis inhibitors was imperative in order to avoid unwanted effects from C5 immediate blockage. Several clinical and preclinical studies have examined in animal models and lately in humans the effects of inhibiting C5a/C5aR1 axis by the investigation of novel peptide drugs against C5aR1. Specifically, avacopan has already received approval in many kidney disorders, such as IgA nephropathy, and may change the therapeutic landscape in DKD [1,18]. In preclinical studies, the use of anaphylatoxin C5a receptor blockers such as PMX53 and PMX205 has also displayed promising outcomes in diabetic animal models [18]. More specifically, administration of these agents has been correlated with considerable decline in albuminuria, inversion in tubulointerstitial fibrosis and glomerulosclerosis, reduction in kidney infiltration by inflammatory cells, as well as transcriptomic restoration of gene expression that modulate kidney metabolic homeostasis [1,17,18]. Of note, as mentioned above, valproic acid administration to streptozotocin-induced diabetic mice reduced albuminuria and glomerulosclerosis through inhibition of diabetes-induced upregulation of complement C5a receptors concomitantly with a reduction in markers of premature aging and senescence-associated secretory phenotype. Thus, valproic acid administration in preclinical studies revealed significant renoprotective effects by reducing pro-fibrotic factors and podocyte apoptosis [30]. Certainly, the safety of C5a receptor blockers in clinical practice is awaited in order to see these spectacular results in the field of DKD.

As regards conventional renoprotective therapies, it seems that the traditional use of RAAS inhibitors does not reduce efficiently the C5a plasma levels [17]. On the contrary, transcriptome array studies revealed an inverse correlation between sodium-glucose transporter protein-2 (SGLT-2) expression with intrarenal C5a synthesis supporting the nephroprotective effects of SGLT-2 inhibitors through mechanisms other than glucose regulation and reduction in intraglomerular pressure [60]. Notably, a recent preclinical study revealed new insights about the renoprotective role of finerenone, a new generation of non-steroidal selective mineralocorticoid receptor antagonist, in DKD. The authors supported that the mechanism by which finerenone improved DKD is based on the modulation of the complement system, specifically by inhibiting the C5a-C5aR1 axis via Gnαi2 downregulation in macrophages, a key downstream component of the C5aR1 pathway [61]. 

## Conclusions

Nowadays, enormous costs are spent worldwide in order to treat the complications of diabetes mellitus. Among these, DKD, one of the most serious vascular consequences of diabetes, remains in the foreground as it consists the leading cause of ESKD. Lately, several studies have made clear a causal relationship between complement cascade activation and DKD. In support of the above, transcriptome-wide genome analyses unveil the involvement of complement system activation in DKD pathophysiology through upregulation of complement pathways and infiltration of its components in both glomerular and tubular human kidney biopsy samples.

The major complement mediator is considered to be anaphylatoxin C5a and various clinical and preclinical studies have shown a causal relationship between C5a/C5aR1 signaling axis and progression of kidney inflammation, injury, and fibrosis. Elevated plasma C5a levels were positively correlated with the degree of proteinuria, while negatively associated with GFR decline in diabetic patients. The most significant confirmed statement is that upregulation of the C5a signaling pathway is proven to precede renal complications in diabetic patients, and its inhibition leads to amelioration of diabetes-associated kidney lesions. More than that, urinary excretion of C5a was proven to be an excellent biomarker of the histological progression of DKD in humans, with almost 80% sensitivity and approximately 70% specificity to predict DN in advanced stages. To conclude, these findings make clear that C5a could be a robust biomarker of DKD progression as well as the epicenter of targeted therapy with novel anti-C5a and C5aR1 antagonists. In the future, complement-targeting drugs, together with finerenone, RAAS and SGLT-2 inhibitors, may succeed even the unfulfilled dream; prevention and targeted therapy of DKD.
